# A simple bridging flocculation assay for rapid, sensitive and stringent detection of gene specific DNA methylation

**DOI:** 10.1038/srep15028

**Published:** 2015-10-13

**Authors:** Eugene J. H. Wee, Thu Ha Ngo, Matt Trau

**Affiliations:** 1 Centre for Personalized NanoMedicine, Australian Institute for Bioengineering and Nanotechnology (AIBN), The University of Queensland, St Lucia, Queensland, Australia; 2 School of Chemistry and Molecular Biosciences, The University of Queensland, St Lucia, Queensland, Australia; 3 Faculty of Biotechnology, Vietnam National University of Agriculture, Hanoi, Vietnam

## Abstract

The challenge of bringing DNA methylation biomarkers into clinic is the lack of simple methodologies as most current assays have been developed for research purposes. To address the limitations of current methods, we describe herein a novel methyl-protein domain (MBD) enrichment protocol for simple yet rapid and highly stringent selection of highly methylated DNA from limiting input samples. We then coupled this with a DNA-mediated flocculation assay for rapid and low cost naked-eye binary evaluation of highly methylated genes in cell line and blood DNA. The low resource requirements of our method may enable widespread adoption of DNA methylation-based diagnostics in clinic and may be useful for small-scale research.

Epigenetic changes in DNA are a potential source of disease biomarkers^1^. One form of DNA epigenetic change is the methylation of the cytosine (5 mC) in cytosine/guanine dinucleotide (CpG), particularly in CpG islands (CGI) of regulatory regions that function to modulate cellular processes^1^. Most approaches however, detect DNA methylation via bisulfite conversion^2^,^3^ of DNA followed by some form of sequencing^4^,^5^,^6^,^7^. To avoid the complications associated with bisulfite-based approaches, affinity capture methods using proteins such as Methyl-Binding Domain (MDB) or antibodies raised against 5 mC have been developed and almost exclusively used with next generation sequencing (NGS) in many recent DNA methylation studies^8^,^9^,^10^. While these methods are excellent for research, simpler and more convenient methods to detect gene-specific methylation for routine diagnostics are still lacking. Although various strategies have been developed with affinity-based approaches^7^,^8^,^9^,^11^,^12^,^13^,^14^ to address clinical needs, all approaches still require some form of an optical readout method or other specialized equipment to evaluate differentially methylated regions (DMRs).

While MBD-based approaches are useful, its specificity is compromised with low amounts of DNA input. Several tedious and time consuming strategies have been proposed to address this limitation to various degrees of success^9^,^12^,^15^. MBD-based assays also tend to have bias towards CpG dense sequences and cannot discern methylation states of individual CpGs^8^,^10^. In addition, quantifying the degree of methylation is challenging and most methods rely on quantitative PCR (qPCR) or NGS as a proxy measure of methylation levels to estimate and validate enrichment^8^,^9^,^10^,^12^,^15^. Nonetheless, a useful characteristic of MBD-enrichment methods is the almost binary specificity of the enzymes under the appropriate salt concentrations^8^,^9^, i.e., either MDB captures methylated DNA or not. This binary characteristic may be useful in situations where significant changes e.g., regions of high differential methylation (HDMRs) that are predictive of disease outcomes in clinic. Therefore, a readout method mirroring these digital yes/no biomarkers may also be useful. A possible binary readout approach could be a DNA polymer-mediated bridging flocculation assay^16^. As flocculation typically occurs abruptly between solution and precipitate phases^17^,^18^,^19^,^20^,^21^,^22^,^23^, marrying MDB enrichment assays with a flocculation-based readout may result in a simple, rapid, low-resource method to evaluate HDMRs. While bridging flocculation was been used to detect pathogen genetic material^16^, there are no methods to-date using bridging flocculation to evaluate DNA methylation status.

Bridging flocculation is a colloid chemistry phenomenon typically used in colloidal separation processes (e.g., to clarify contaminated water). The phenomenon has been described in detail by Ruehrwein, R.A.^17^, La Mer and Healy^18^,^19^,^20^ to be the result of the reversible surface adsorption of polymers which are long enough to cross-link multiple particles together and thus flocculate out of solution. This reversible adsorption is a distinguishing feature which differentiates a bridging flocculation assay from various previously described nanoparticle-based assays^24^,^25^,^26^,^27^ which are based on irreversible aggregation of particles to evaluate assay outcomes. An advantage of the reversibility of bridging flocculation is the added versatility to “tune” the assay possibly for specific applications.

The key requirement of a bridging flocculation assay is the ability to discriminate between long and short DNA polymer segments. This is enabled by adjusting the solution conditions (e.g., salt concentration and pH) such that polymer/surface interactions are stronger than the polymer/solution interaction (as defined by the relevant Flory-Huggins Parameters^21^,^22^,^23^). Under, such conditions longer polymer chains (DNA amplicons) will displace surface adsorbed shorter polymers (primers) to induce a spontaneous flocculation.

Since bridging flocculation is easily seen with the naked eye, it is also a very attractive low resource evaluation system compared to conventional methodologies and may be useful for low cost routine diagnostics. In contrast to other nanoparticle-based approaches^24^,^25^,^26^,^27^ where the colour shifts are usually subtle and require spectrometry for verification, the shift from cloudy to a clear solution in a flocculation assay is of better visual contrast and thus more easily evaluated by naked eye without additional equipment. Herein, we first describe a method using a novel MBD protocol to selectively enrich for methylated DNA from limiting samples. This was then followed by a robust isothermal recombinase polymerase amplification (RPA)^28^ to rapidly generate large amounts of HDMR-specific DNA polymers. Finally, the presence of the amplified HDMR is evaluated by naked eye via a DNA-mediated bridging flocculation assay. Only nanogram amounts of starting genomic material was required and the assay was sensitive to 10% changes in methylation under current conditions. The assay was completed in under two hours and required only minimal equipment, i.e, pipettes, a heating block and a magnet. Finally, the assay was applied to a panel of cells and whole blood-derived DNA to test for the presence of three potential cancer-related HDMRs. We believe the speed, simplicity and low resource requirement of the method could have broad DNA methylation-based applications in the clinic.

## Results and Discussion

### The MBD flocculation assay

**Figure 1:**
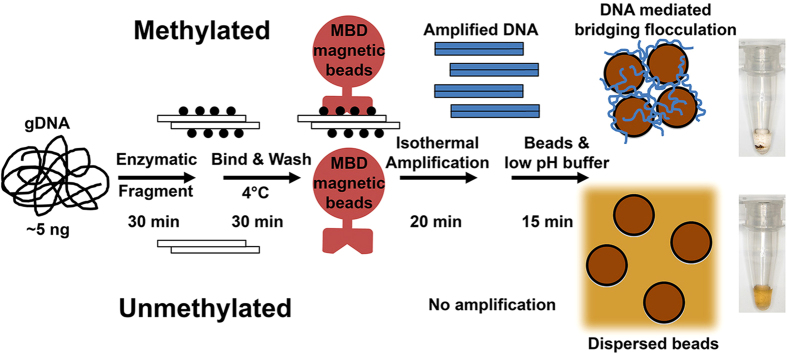
Schematic of the assay. (**1**) Genomic DNA is enzymatically fragmented to sizes compatible with MBD enrichment. (**2**) Rapid high stringency enrichment of methylated DNA is performed on ice (4 °C). (**3**) Regions of interests are amplified via an isothermal method. (**4**) The presence of long amplified DNA induces a bridging flocculation which indicates the presence of the HDMR of interest.

To realise the method (Fig. 1), we first enzymatically fragment 5 ng of genomic DNA (gDNA) for the MBD enrichment. We chose enzymatic fragmentation of input DNA because it was an established and robust molecular technique that required only a heating block unlike physical shearing by sonication which may be time consuming and requires specialized equipment. The combination of the MseI and MluCI restriction enzymes (recognition sequence: TTAA and AATT respectively) were selected because they cut frequently in the human genome to generate fragments compatible with MBD enrichment while avoiding excessive fragmentation of CGIs where HDMRs are typically found. Next, a novel protocol using MBD coupled to magnetic beads were then introduced to select for HDMRs. Specific HDMRs are then amplified with RPA. If the HDMR of interest is methylated, RPA amplicons are generated and is able to initiate a DNA-mediated flocculation of beads resulting a clear solution.

### Simple, rapid and highly stringent MDB enrichment

**Figure 2:**
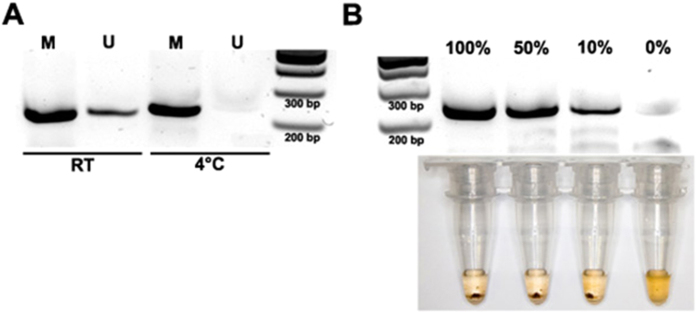
(**A**) Gel electrophoresis image of RPA products using ESR1 primers demonstrating improved stringency of MDB enrichment at low temperatures (4 °C) without a lost in performance as compared to the assay performed at room temperature (RT). M: methylated control, U: unmethylated control. (**B**) Top: Gel electrophoresis image of RPA reactions using ESR1 primers for DNA inputs at various levels of methylation. Bottom: Photos showing flocculation occurring from as little as 10% methylated samples.

Relatively high salt (300 mM) buffers^9^ have been found to improve the stringency of MBD enrichment. However, current protocols require long incubations. To enable rapid yet high stringency enrichment we performed both the binding and washing steps on ice (4 °C). The purpose of using a low temperature assay is to reduce the enzyme kinetics such that only high affinity interactions may proceed effectively. Using primers against the CGI associated with the ESR1 gene promoter, a known HDMR in breast cancer^29^, we first tested stringency of our modified MBD enrichment step using 5 ng of whole genome amplified (WGA) DNA as an unmethylated control and 5 ng *in vitro* methylated WGA DNA as the methylated control. As indicated by subsequent RPA amplification of enriched DNA, performing the whole MBD assay (binding and washing) on ice resulted in highly specific enrichment of only methylated DNA with minimal loss in performance in about 30 minutes (Fig. 2A). This was a significant improvement in both speed and specificity over protocols previously described^9^,^12^,^15^ where either cocktails of different MDB enzymes and/or overnight incubations were needed to achieve high stringency with low input. Moreover, the robust and rapid isothermal amplification used here further simplified and hastened the assay.

### Assay sensitivity and specificity

As naked-eye evaluations may be useful for binary diagnostic outcomes such as MBD-enriched HDMRs, we used a flocculation assay to evaluate amplification. As only minimal equipment is needed for evaluation, a flocculation assay may also be suitable for low resource settings. Following RPA amplification for HDMRs of interest we used, as a proof-of-concept, the solid phase reversible immobilization (SPRI)^30^ carboxylic-coated magnetic beads for DNA purification by precipitating DNA onto the surface of the beads in a high salt PEG buffer (see Methods section for details). Then instead of eluting the bound DNA, a low pH buffer was introduced to trigger a flocculate only if high amounts of RPA amplicons with sufficient lengths were present^16^, which in turn represents the presence of the MBD-enriched HDMR. If no amplification occurred, indicating a lack of methylation, the 1 μm beads readily disperse into solution due to the charge repulsion of exposed carboxylic acid groups on the bead surface. To our knowledge, this is a first bridging flocculation assay for a MBD/RPA-based approach.

Confident that we could now rapidly detected HDMRs with high stringency, we turned our attention to determining the detection limits of the assay, specifically (1) the minimum input needed to generate enough RPA amplicons to trigger a flocculate and (2) the minimum detectable amount of methylated DNA. To this end, we processed 5 ng of gDNA titrated to various levels of methylation (100%, 50%, 10% and 0%). We could robustly detect the presence of methylated DNA from as little as 10% of the total DNA input on both gel electrophoresis visualization and with the flocculation assay (Fig. 2B). This also suggested that a minimum of 0.5 ng (10% methylated sample) of starting methylated DNA was required for a detectable signal. WGA generated control DNA used in this study was generated by the multiple strand displacement method^31^ and hence may be partially fragmented and/or single stranded. This may in turn introduce some over (or under)-estimations of the amount sample input. However, since the same batch of WGA was used in the methylation controls, any bias in methylation ratios was unlikely. Thus, considering the data, we were confident that our approach was both specific and sensitive to highly methylated DNA in the low nanogram regime.

Whilst quantification is normally done in methylation studies, a limitation of MBD-based enrichment assays is the difficulty in quantifying methylation levels. Hence exploiting MBD’s binary selection for highly methylated sequences can potentially avoid the need for quantification. We therefore believe that the method could be suited for detecting HDMR biomarkers that are completely unmethylated in normal tissue. Nonetheless (semi)quantification may be achieved by evaluating different MBD elution fractions^8^,^10^ for the presence of a flocculate as a proxy estimate for methylation levels. Another known limitation of MBD-based assays is the bias towards highly CpG dense regions^8^,^10^. However, this characteristic of targeting HDMRs with high CpG density, i.e. a binary methylation state, is ideal for a similarly discrete binary readout of a flocculation assay.

Recently an on-chip MBD/RPA approach using DNA-induced shifts in magnetic resonance^11^ was described. While rapid and useful, the approach requires auxiliary equipment to evaluate the presence of RPA amplicons. In contrast, results are easily evaluated with the naked eye by our approach within a similar timeframe and with less starting material. In addition, we have also demonstrated the ability of our method to detect low level (10%) methylation, albeit qualitatively, and from nanogram amounts of DNA input.

### Detecting HDMRs in cells and blood

**Figure 3:**
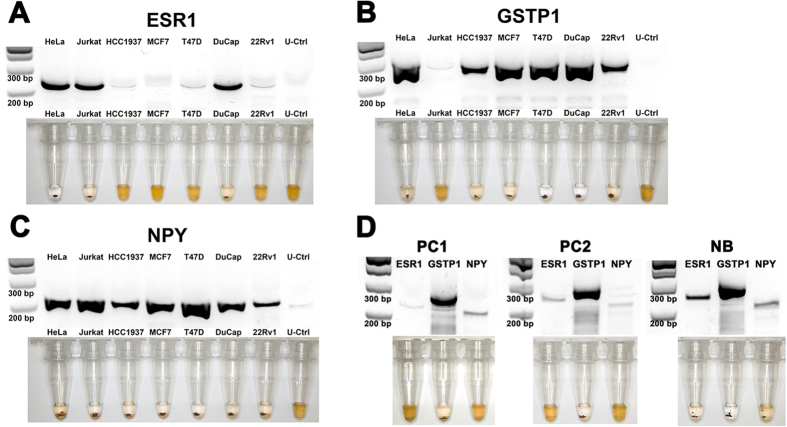
Methylation profiles of 7 human cancer cell lines for (**A**) ESR1, (**B**) GSTP1 and (C) NPY. Unmethylated controls (U-Ctrl) was included to validate assay stringency. (**D**) Whole blood methylation profiles of ESR1, GSTP1 and NYP from two prostate cancer samples (PC1 and PC2) and normal blood (NB) DNA pooled from 25 female donors. Top: Gel electrophoresis image of RPA reactions. Bottom: Photos showing flocculation as proxy for methylation states.

Finally, to demonstrate an application on more complex biological samples, we extended our assay to evaluate the methylation status of two additional potential biomarkers GSTP1^32^,^33^,^34^ and NPY^35^,^36^,^37^ in addition to ESR1 on a panel of human cancer cell lines (Fig. 3A–C). To control for stringency, unmethylated WGA DNA was also assayed simultaneously with the cell line-derived DNA. As expected, for all three biomarker assays, negligible RPA amplification was detected in the unmethylated controls thus validating assay stringency. In addition, we successfully profiled the methylation states of ESR1, NPY and GSTP1 for all 7 cell line samples. In addition to being also consistent with the literature^38^,^39^, methylation states presented here also were subsequently validated with qPCR (Fig. S1) and bisulfite sequencing (Fig. S2), thus underscoring the accuracy of our approach. Finally, we the applied the assay to two samples of whole blood derived gDNA obtained from men with highly metastatic prostate cancer and one from a pooled sample of 25 normal females (Fig. 3D). For the two prostate cancer samples (PC1 and PC2), highly methylated GSTP1 was detected but not for ESR1 and NPY based on the presence of a flocculate. For the pooled normal DNA sample (NB), all three biomarkers were methylated as indicated by the flocculation assay. While the methylation profiles were unexpected for the normal DNA sample, further investigation is outside the scope of this report. However the data suggests the importance of selecting the appropriate sampling source for specific methylation biomarkers. Although an in-depth study is still needed to fully evaluate clinical utility, the limited number of blood biopsies shown here may be sufficient to demonstrate proof-of-concept and clinical potential. Finally, the assay in its current form, has a lower detection limit of 10% methylation from 5 ng of starting material, hence is not able to detect the low (~1%) copies of methylated cell-free DNA typical of disseminated disease. However, given that RPA can have a sensitivity of ≤10 copies^28^, we believe that better optimized RPA assays could eventually meet this requirement in the near future. Nonetheless, the results here demonstrate the feasibility of our assay with bodily fluids (e.g. whole blood) typically used in routine diagnostics.

In conclusion, we describe for the first time a rapid MBD-based method for stringent enrichment of methylated DNA from low nanogram amounts of input. Coupling this with RPA, we could achieve sensitive detection from at least 10% methylated samples. Positive MBD/RPA results are then evaluated by naked eye with a simple flocculation assay. This is also the first demonstration of a classical colloid chemistry phenomena for methylation detection. The method was also extended to cell line and whole blood derived samples to demonstrate its feasibility as a diagnostic tool. Finally, the use of a flocculation assay to evaluate positive MBD/RPA results enables a label-free, minimal equipment approach for low resource applications that may be beneficial for routine diagnostics and small-scaled DNA methylation research.

## Methods

### DNA sample preparation

WGA DNA was generated using the REPLI-g UltraFast Mini kit (Qiagen) and purified using the DNeasy Blood and Tissue kit (Qiagen). An aliquot of WGA DNA was then treated with SssI methyltransferase overnight and purified with SPRI to generate highly methylated genomic DNA. Cell line derived DNA was also purified with the DNeasy Blood and Tissue kit. Cell lines were purchased from ATCC and cultured according to the manufacturer’s recommendation.

Ethics approval was obtained from The University of Queensland Institutional Human Research Ethics Committee (Approval No. 2004000047) and informed consent was obtained from all subjects prior to collecting blood samples. Methods pertaining to blood samples were carried out in accordance with approved guidelines. Whole blood DNA from prostate cancer patients were purified using a modified SPRI protocol^30^. Briefly, 60 μL of whole blood preserved with EDTA was incubated with 10 μL of proteinase K solution (New England Biolabs, NEB), 60 μL of lysis buffer (3 M Guanidine HCl, 2% Triton X) and 2 μL of RNase A solution (4mg/mL) for 10 minutes at room temperature. 10 μL of carboxyl coated magnetic beads (Thermo Fisher) and 240 μL NaCl/PEG binding buffer (100 mM Tris-HCl, 2.5 M NaCl, 20% w/v PEG8000, pH 8.0) was then added to the lysed blood. After a 10 min incubation at room temperature, DNA bound magnetic beads were collected with a magnet and washed once with a wash solution (400 mM Guanidine HCl, 70% ethanol), twice with 70% ethanol and finally eluted in 30 μL of water. Both blood samples were processed within 24 hours of collection.

To generate DNA fragment sizes compatible with MDB enrichment, MseI and MluCI restriction enzymes (NEB) were used. Briefly, 5 ng of DNA was digested with 1 unit of each enzyme in a 10 μL reaction at 37 °C for 30 minutes. After digestion, 2 μL of the reaction was used for each MBD enrichment.

### Rapid high stringency MBD enrichment

The Epimark Methylated DNA Enrichment kit (New England Biolabs) was used with major modifications to the recommended instructions. Briefly, 2 μg MDB2a-Fc protein was incubated with 10 μL of Protein A magnetic beads at room temperature for 15 minutes. 0.5 μL of the MBD/magnetic bead mix was then used for each 50 μL enrichment reaction in a 1× MBD buffer with NaCl modified to a concentration of 300 mM. DNA was reacted with the MBD/magnetic beads for 15 mins on ice. This was then followed by three 5 minute washes with cold (4 °C) 1× MBD buffer with NaCl modified to 300 mM to remove excess and weakly bound DNA. Enriched DNA was then eluted with 5 μL of 2.5 M NaCl solution. Finally, enriched DNA was purified with SPRI^30^ magnetic beads in NaCl/PEG buffer, washed once with 70% ethanol and eluted in 6 μL of water. 1 μL purified DNA was then used for each RPA reaction.

### RPA amplification

RPA reactions were performed using the TwistAmp Basic Kit (TwistDx) with some modifications to the recommended protocol. Briefly, the modified protocol included 500 nM primers (Table 1) used in each 12.5 μL reaction supplemented with 7 mM MgOAc. After a rapid 15 minute RPA amplification at 37 °C on a heating block, 3 μL was electrophoresed on a gel to verify amplification. The remaining was subjected a SPRI^30^ clean-up to remove RPA reaction components that could interfere with the downstream flocculation assay. Purified amplicons were then eluted in 9.5 μL water.

### Flocculation assay

To perform the flocculation assay, 3 μL of purified RPA amplicons were incubated with 6 μL of SPRI^30^ magnetic beads in NaCl/PEG buffer (100 mM Tris-HCl, 2.5 M NaCl, 20% w/v PEG8000, pH 8.0) at room temperature for 5 minutes. Beads were then captured with a magnet and the supernatant was removed. 20 μL flocculation buffer (200 mM sodium acetate buffer, pH 4.4) was then added immediately and allowed to incubate for an additional minute on the magnet. Finally, tubes were then removed from the magnet and agitated by gently tapping the sides of the tubes. Positive tests resulted in a flocculate of DNA/bead pellet while beads readily redispersed into solution for negative tests.

## Additional Information

**How to cite this article**: Wee, E. J. H. *et al.* A simple bridging flocculation assay for rapid, sensitive and stringent detection of gene specific DNA methylation. *Sci. Rep.* **5**, 15028; doi: 10.1038/srep15028 (2015).

## Supplementary Material

Supplementary Information

## Figures and Tables

**Table 1:** Primer sequences used in this study.

Gene Target	5′-Fwd-3′	5′-Rev-3′	Genome Coordinates UCSC HG19	#CpGs	Size (bp)
ESR1	GTTCGTCCTGGGACTGCACTTGCTCCCGTC	AGATGCTTTGGTGTGGAGGGTCATGGTCATGGT	chr6: 152128831–152129077	24	247
GSTP1	CATCCTCCCCCGGGCTCCAGCAAACTTTTCTTT	AAACAGGTTCCTCCGAAGATTTCACACAACACT	chr11: 67351561–67351840	15	280
NPY	TGAGTTACCTTTTAGCAGATATGGAGGGAGAAC	CAAAGAGATTTGGAGCCCAAGAATCCAGGGAG	chr7: 24324146–24324355	14	210
