# Supplementary material for: A simple bridging flocculation assay for rapid, sensitive and stringent detection of gene specific DNA methylation
Source: Sci Rep. 2015 Oct 13;5:15028. doi: 10.1038/srep15028 (PMC4602207; doi:10.1038/srep15028)
Supplement: Supplementary Information [file srep15028-s1.pdf]

Supplemental Data for

A simple bridging flocculation assay for rapid, sensitive and stringent detection of gene specific DNA methylation

Eugene J. H. Wee[1,*], Thu Ha Ngo[1, 2] and Matt Trau[1, 2,*]

Gene Target	Fwd	Rev	Genome Coordinates UCSC HG19
ESR1	CCTGGGACTGCACTTGCT	AGGGCAGAAGGCTCAGAAAC	chr6: 152128837-152129008
NPY	AGCAGATATGGAGGGAGAACC	AGAGATTTGGAGCCCAAGAA	chr7: 24324159-24324352
GSTP1	GGCTCCAGCAAACTTTTCTTT	GATAAGGGGGTTCGGATCTC	chr11: 67351573-67351733

Table S1: Primers used for quantitative PCR (qPCR) validation of MBD enrichment.

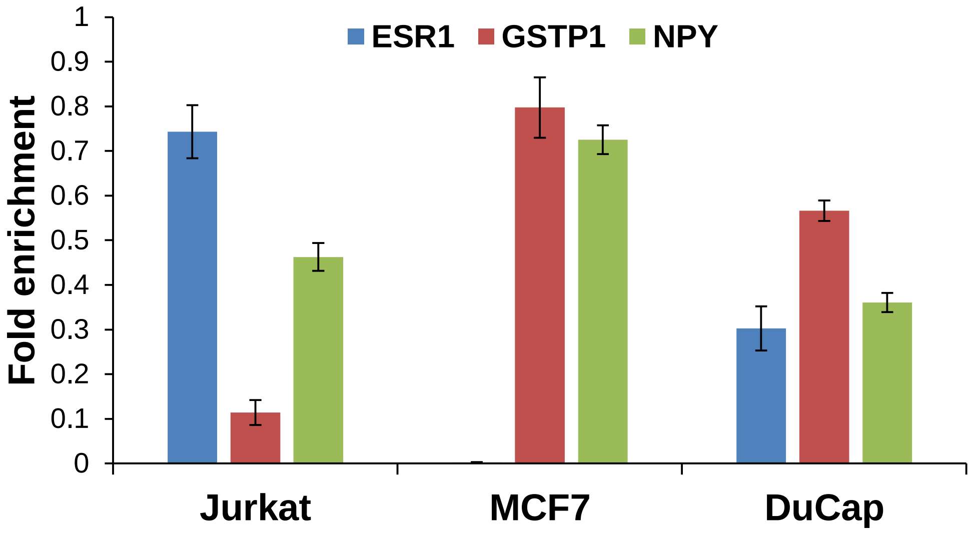

Figure S1. qPCR validation of methylation states in Jurkat, MCF7, and DuCap cell lines for the ESR1 (blue), GSTP1 (red) and NPY (green). Error bars represent standard deviation of 4 biological replicates. qPCR was performed using Kapa2G Fast Hotstart Readymix (KapaBiosciences) supplemented with 250nM of each primer, 1 µL of DNA from either the MBD captured fraction or MBD input. Thermo-cycling was done on the ABI 7500 Fast Real Time PCR platform (Applied Biosystems) with the following program: 95°C for 2 minutes followed by 35 cycles of 95°C for 15s, 60°C for 30s and 72°C for 30s. Fold Enrichment was estimated by following formula:

$$\begin{aligned} Enrichment &= MBD \div input \\ &= 2^{-Ct_{MBD}} \div 2^{-Ct_{input}} \\ &= 2^{-(Ct_{MBD}-Ct_{input})} \end{aligned}$$

Where Ct is the cycle threshold.

Gene Target	Fwd	Rev	Genome Coordinates UCSC HG19
**ESR1**	GTTYGTTTTGGGATTGTATTTGTTTT	AAATACTTTAATATAAAAAATCATAATCATAA	chr6: 152128835-152129077
**NPY**	TTTTAGTAGATATGGAGGGAGAAT T	AAACCCAAAAATCCAAAAAAATAACA	chr7: 24324155-24324343
**GSTP**	GTTGGGGTTGTAGTTTATAGTTTTT	AATTCCTCCAAAAATTTCACACAA	chr11: 67351678-67351835

Table S2: Primers used for bisulfite sequencing.

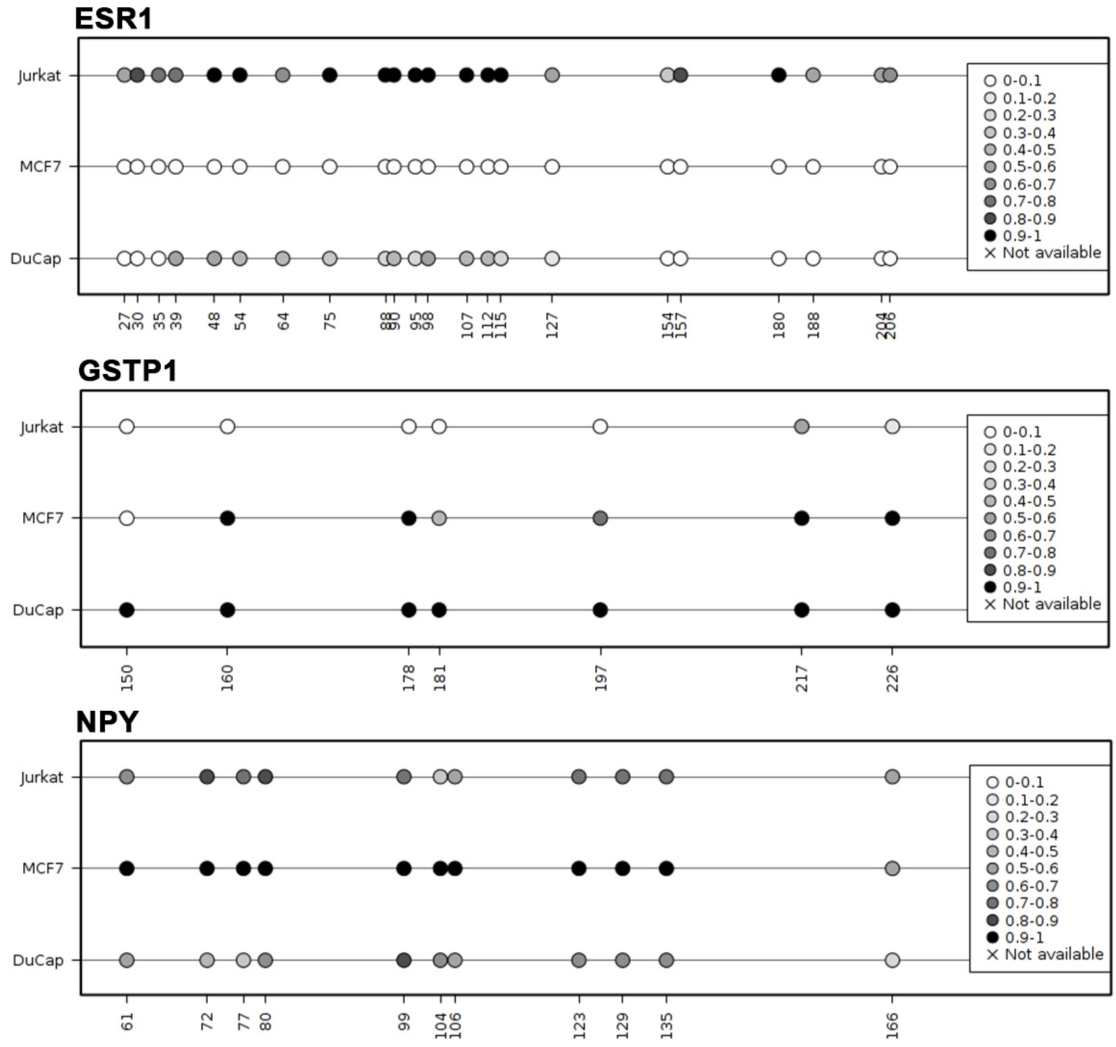

Figure S2. Bisulfite sequencing of Jurkat, MCF7, and DuCap cell lines for the ESR1, GSTP1 and NPY. Individual CpGs are represented by circles and methylation levels are indicated by the degree of shading. Numbers below each circle is the nucleotide position in the respective RPA amplicons described in the main text.
